# Down Regulation of CIAPIN1 Reverses Multidrug Resistance in Human Breast Cancer Cells by Inhibiting MDR1

**DOI:** 10.3390/molecules17067595

**Published:** 2012-06-20

**Authors:** Dan Lu, Zhibo Xiao, Wenxiu Wang, Yuqing Xu, Shujian Gao, Lili Deng, Wen He, Yu Yang, Xiaofei Guo, Xuemei Wang

**Affiliations:** 1 Department of Oncology, the Second Affiliated Hospital of Harbin Medical University, Harbin 150086, China; 2 Department of Plastic Surgery, the Second Affiliated Hospital of Harbin Medical University, Harbin 150086, China

**Keywords:** breast cancer, multidrug resistance, CIAPIN1gene, MDR1gene, RNA interference

## Abstract

Cytokine-induced apoptosis inhibitor 1 (CIAPIN1), initially named anamorsin, a newly indentified antiapoptotic molecule is a downstream effector of the receptor tyrosine kinase-Ras signaling pathway. Current study has revealed that CIAPIN1 may have wide and important functions, especially due to its close correlations with malignant tumors. However whether or not it is involved in the multi-drug resistance (MDR) process of breast cancer has not been elucidated. To explore the effect of CIAPIN1 on MDR, we examined the expression of P-gp and CIAPIN1 by immunohistochemistry and found there was positive correlation between them. Then we successfully interfered with RNA translation by the infection of siRNA of CIAPIN1 into MCF7/ADM breast cancer cell lines through a lentivirus, and the expression of the target gene was significantly inhibited. After RNAi the drug resistance was reduced significantly and the expression of MDR1mRNA and P-gp in MCF7/ADM cell lines showed a significant decrease. Also the expression of P53 protein increased in a statistically significant way (*p* ≤ 0.01) after RNAi exposure. In addition, flow cytometry analysis reveals that cell cycle and anti-apoptotic enhancing capability of cells changed after RNAi treatment. These results suggested CIAPIN1 may participate in breast cancer MDR by regulating MDR1 and P53 expression, changing cell cycle and enhancing the anti-apoptotic capability of cells.

## 1. Introduction

Breast cancer is one of the common cancers all over the World [1]. There are about 1,200,000 women suffering from breast cancer around the World, 500,000 of which die of this disease every year. In China, incidence of breast cancer tends to increase year by year [2]. Breast cancer often has a poor survival rate due to late clinical presentation and rapid progression. Although recent improvements in early detection, surgical techniques and chemoradiotherapy have effectively promoted advancements in its treatment, its overall survival rate is still low [3]. Due to the importance of chemotherapy in treating breast cancer, the development of multidrug resistance (MDR) becomes a serious obstacle to effective chemotherapy [4]. Although the molecular mechanism(s) of the MDR have not yet been elucidated in breast cancer cells, some studies have reported that the mechanisms of MDR were close associated with the overexpression of P-glycoprotein (P-gp) encoded by MDR1 gene in tumor cells [5,6,7]. Therefore, the inhibition of P-gp expression in tumor cells could be one of the most effective ways to reverse MDR and make tumor cells resensitize to chemotherapy [8].

Cytokine induced apoptosis inhibitor 1 (CIAPIN1) is a novel antiapoptotic molecule. Compared with the Bcl-2 family, caspase family and signal transduction molecules, it is a distinct apoptosis regulatory molecule and it affects the RAS signaling pathway as a mediator [9]. Most importantly, it plays a vitally important role in malignant phenotypes of some cancers. Some previous studies reported that CIAPIN1 could affect the cell proliferation and cell cycle progression and it may be involved in regulating MDR in some cancers [10,11,12,13,14,15,16]. However, until now no article has reported whether CIAPIN1 is associated with the mechanism of MDR in breast cancer cells or not. These findings lead to the hypothesis that CIAPIN1 may influence the MDR of breast cancer cells through cell cycle regulation, apoptosis and the expression of the MDR1 gene. The aim of this study was to explore the relationship between CIAPIN1 and MDR of breast cancer cells. To our knowledge, this article is the first to report an evaluation of CIAPIN1 in reversing MDR of breast cancer cells 

## 2. Results and Discussion

### 2.1. Results

#### 2.1.1. Expression and Relationship of P-gp, CIAPIN1 and p53 Protein in Breast Cancer Patient Samples and Cell Lines

**Figure 1:**
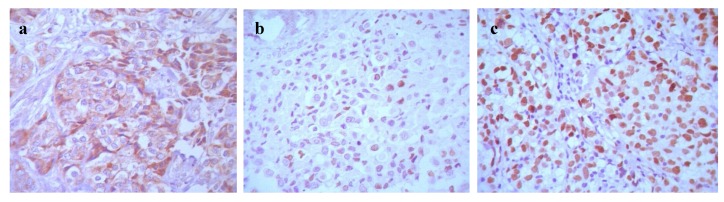
Immunohistochemical analysis of P-gp, CIAPIN1 and P53 protein expression in breast cancer tissues (41 cases): (**a**) expression of P-gp (SP, ×400); (**b**) expression of CIAPIN1 (SP, ×400); (**c**) expression of P53 (SP, ×400).

To discuss the relationship of P-gp, CIAPIN1and p53 primarily in breast cancer, immunohistochemistry was performed on 41 breast cancer tissue samples following the manufacture’s instruction. P-gp expression had a positive rate of 51.2% (21/41), CIAPIN1 of 34.1% (14/41), P53 of 41.5% (17/41), which had a significant difference comparing with the normal breast tissue around the cancer (*p* < 0.05). P-gp protein was expressed in cytoplasm and cell membrane, while CIAPIN1 was in the cell nucleus (Figure 1). The P-gp expression showed a positive correlation with the expression of CIAPIN1 (*p* < 0.01) and of P53 (*p* < 0.025) (Table 1). Immunohistochemistry tests thus implied that CIAPNI1 and p53 participated in multi-drug resistance in breast cancer, and that it might participate through regulation of P-gp expression. 

**Figure 2:**
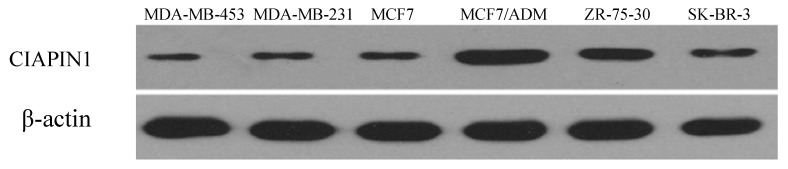
Expression of CIAPIN1 protein in breast cancer cell lines by Western blot. There was the significant difference in the expression level of CIAPIN1 protein between MCF7 and MCF7/ADM cells.

To investigate the differences of CIAPIN1 levels in various breast cancer cell lines, we analyzed by Western blot the amount of CIAPIN1 protein. Results showed the significant increase of CIAPIN1 protein level in MCF7/ADM cells possessing MDR properties compared with MCF7 cells (Figure 2). In other words, there was the significant difference in the expression level of CIAPIN1 protein between MCF7 and MCF7/ADM cells.

#### 2.1.2. Identification of Recombined Plasmid of siRNA Targeting CIAPIN1 and Selection of the Best Interference siRNA Sequence

**Figure 3:**
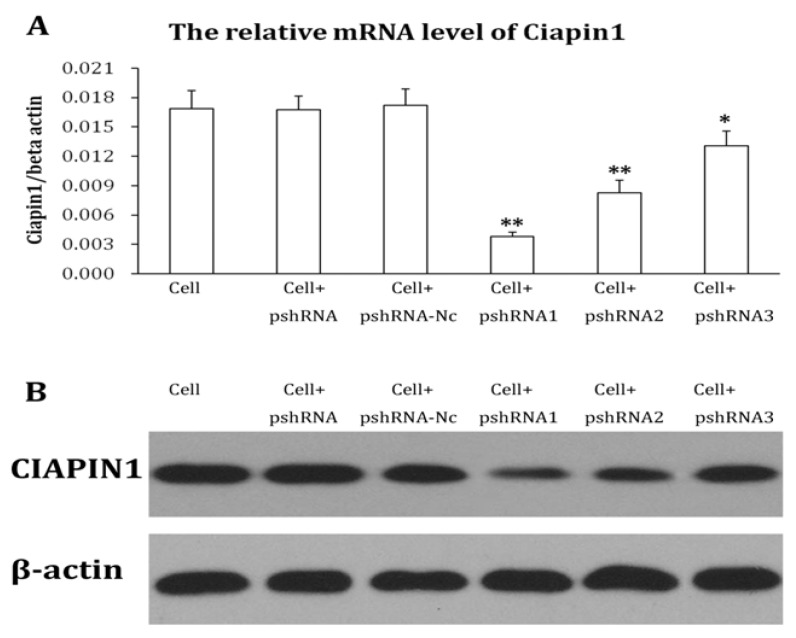
Expression of CIAPIN1 mRNA and protein in each cell line after transfection. Among three siRNAs targeting CIAPIN1, CIAPIN1mRNA and protein expression are most low in CIAPIN1-siRNA1 which is the best interference siRNA sequence. (**A**) Expression of CIAPIN1 mRNA in each cell line after transfection by Real Time-PCR; (**B**) Expression of CIAPIN1 protein in each cell line after transfection by Western blot.

DNA sequencing was conducted on every siRNA and the sequences were proved to be the same as designed, which proved the oligonucleolide fragments were successfully inserted into the pSIH1-H1-copGFP vector. We transiently transfected recombined plasmid into 293 cells and observed cell growth condition with a fluorescent microscope. In the fluorescent visual field, we observed transfection efficiency was 90% 48 h after transfection. Among three siRNAs targeting CIAPIN1, CIAPIN1mRNA and protein expression are the lowest in CIAPIN1-siRNA1 which is the best interference siRNA sequence and was chosen for use in the next experiment (Figure 3).

#### 2.1.3. RNA Interference Test by Lentiviral-Based Vector

**Figure 4:**
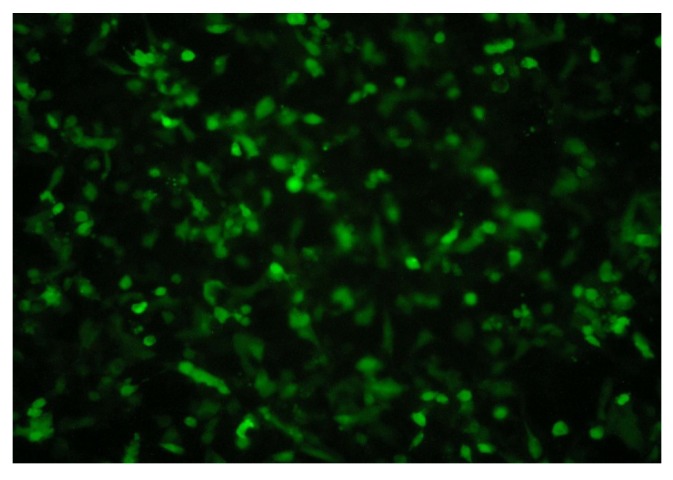
Fluorescence microscopy result of MCF7/ADM 96 h after lentiviral infection (MOI = 6 × 120). The condition of lentiviral infected cells is good and the percent of cells expressing green fluorescent protein is more than 90%.

**Figure 5:**
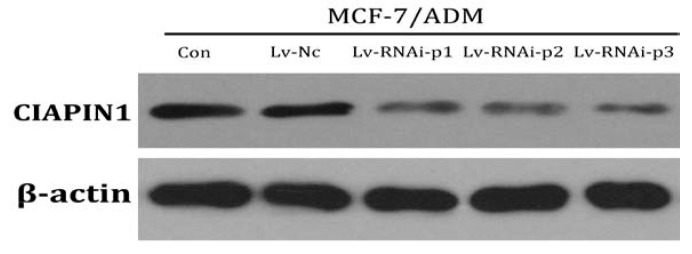
We infected lentiviral expressed vector into MCF7/ADM cells (MOI = 6) and obtained high inhibition efficiency of protein expression (>83%) while the expression lever of target gene had no change in various clone ages.

We infected lentiviral expressed vector into MCF7/ADM cells (MOI = 6) and obtained a stably expressed cell line after screening. The gene CIAPIN1 silencing efficiency is 88% as detected by Real Time-PCR while the inhibition efficiency of protein expression level is 83% by Western blot, which demonstrated that we successfully accomplished RNA interference targeting CIAPIN1 through the lentivirus. We also found the expression of Green Fluorescent Protein (GFP) and target gene showed no change at various clone ages, which proved the MCF7/ADM-CIAPIN1 RNAi cell line is of good quality (Figure 4 and Figure 5).

#### 2.1.4. CIAPIN1siRNA Reversing the Multidrug Resistance

We measured the IC_50_ values of MCF7/ADM cells exposed to several clinical chemotherapeutics (epirubicin, paclitaxel, gemcitabine) and observed that they decreased significantly after RNAi to the same extent as MCF7 cells (Table 2). The drug resistance was reduced significantly. We concluded that RNA interference targeting CIAPIN1 can reverse the MDR properties of breast cancer efficiently. 

#### 2.1.5. CIAPIN1siRNA Down Regulate Expression of MDR1

**Figure 6:**
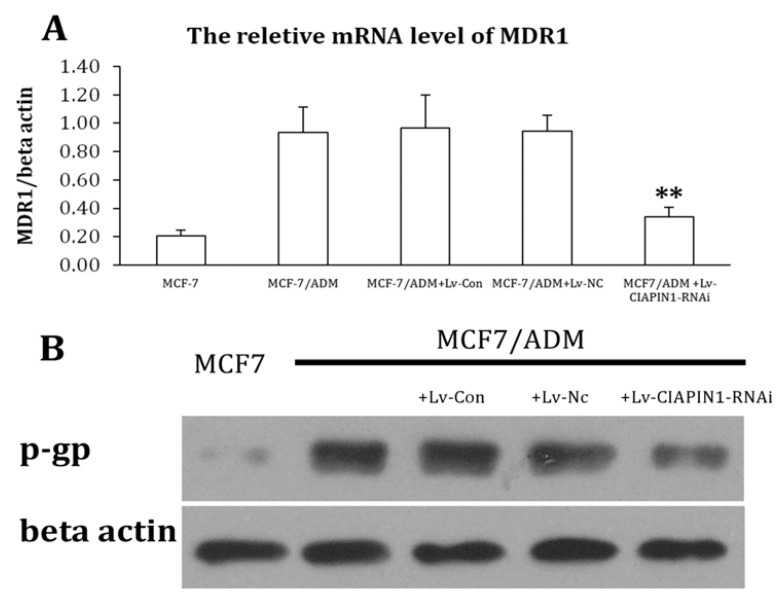
The expression of MDR1mRNA and P-gp in MCF7/ADM cell lines had a statistical significance before and after RNAi (*p* ≤ 0.01). These results indicated that CIAPIN1 may affect MDR-1 to inhibit P-gp expression in drug-resistant MCF7/ADM cell. (**A**) Expression of MDR1 mRNA in breast cancer cell line by Real Time-PCR; (**B**) Expression of MDR1 mRNA in breast cancer cell line by Western blot.

**Figure 7:**
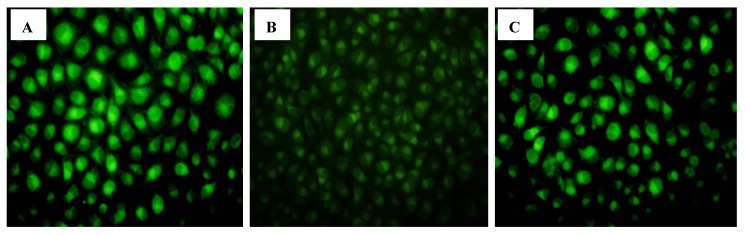
Fluogram of various breast cancer cell line stained by Rhodamine 123 (×400). (**A**): MCF7; (**B**): MCF7/ADM; (**C**): MCF7/ADM after RNAi.

To investigate the effect of CIAPIN1 on MDR1 expression of breast cancer cells, MDR1mRNA and P-gp of MCF7/ADM cell were determined by Realtime PCR and Western blot, respectively, under CIAPIN1 gene silencing conditions. As expected, the expression of MDR1mRNA and P-gp in MCF7/ADM cell lines displayed a statistical significance before and after RNAi (*p* ≤ 0.01) exposure. These results indicated that CIAPIN1 may affect MDR-1 to inhibit P-gp expression in drug-resistant MCF7/ADM cells (Figure 6). We got a similar result in a Rhodamine 123 staining test (Figure 7). The fluorescence intensity of MCF7/ADM is distinctly lower than its parental cell line MCF7, which shows that the drainage ability is stronger in the drug resistant cell line, while in MCF7/ADMsiRNA cell line the fluorescent intensity was enhanced remarkably, which means the drainage ability is weakened after RNAi exposure when CIAPIN1 is silenced. 

#### 2.1.6. Cell Cycle Analysis

**Figure 8:**
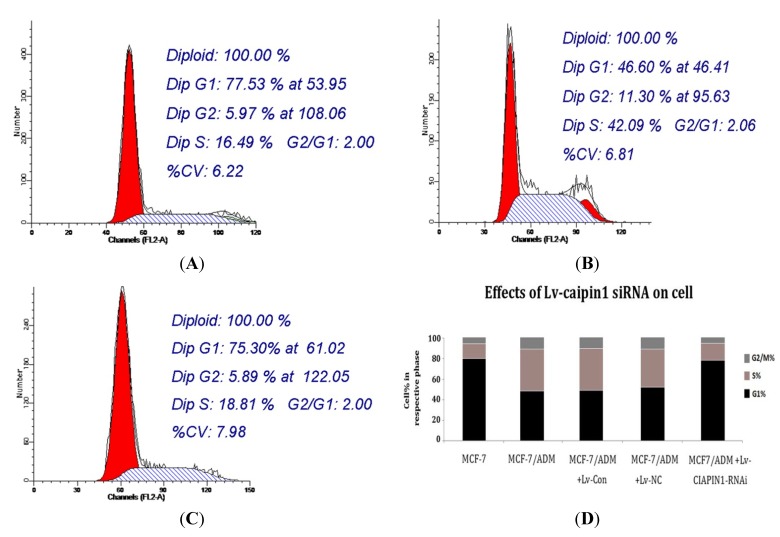
Results from flow cytometry analysis showed that the cell cycle distribution was significantly affected by siRNA targeting CIAPIN1. (**A**): MCF7; (**B**): MCF7/ADM before RNAi; (**C**): MCF7/ADM after RNAi; (**D**): Proportion of cell cycle of various breast cancer cell lines.

Our flow cytometry analysis results showed that the cell cycle distribution was significantly affected by siRNA targeting CIAPIN1. The cell cycle profile indicates that the number of cells in G1 phase increased markedly from (46.60 ± 4.27)% before RNAi to (75.30 ± 4.80)% after RNAi, while those in S phase decreased from (42.09 ± 6.04)% to (18.81 ± 5.18)% (Figure 8). These results suggested that siRNA targeting CIAPIN1 inhibited the entry of cells into S phase, hence indicating that CIAPIN1 exerted a promoting effect on cell cycle progression which might partially participate in the MDR process.

#### 2.1.7. Apoptosis Analysis

**Figure 9:**
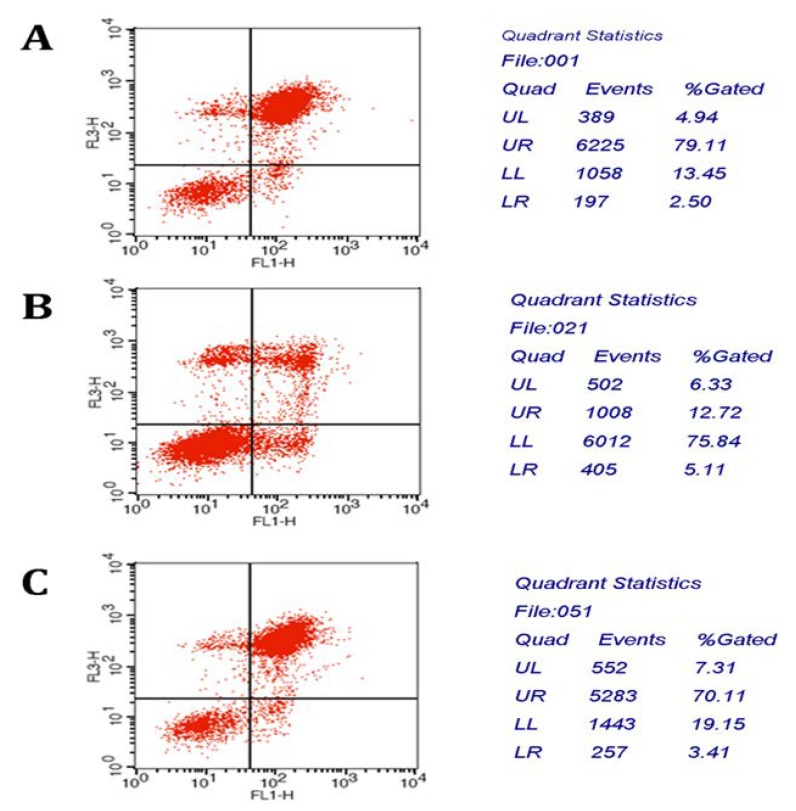
Apoptosis of cells were determined by flow cytometry analysis. Compared with the cells without the treatment of CIAPIN1 siRNA, the apoptosis rate significantly increased after RNAi. (**A**): MCF7; (**B**): MCF7/ADM before RNAi; (**C**): MCF7/ADM after RNAi.

We postulated that the effect of CIAPIN1siRNA on MCF7/ADM was related to its impact on apoptosis. To test this assumption, we stably transfected CIAPIN1siRNA into MCF7/ADM cells. Apoptosis of cells were determined by flow cytometry analysis. Compared with the cells without the CIAPIN1 siRNA treatment, the apoptosis rate increased significantly from (17.83 ± 2.24)% before RNAi to (73.52 ± 7.95)% after RNAi (Figure 9). These results demonstrated that CIAPIN1 siRNA could induce apoptosis of MCF7/ADM cells.

#### 2.1.8. CIAPIN1siRNA Influenced the Expression of P53

**Figure 10:**
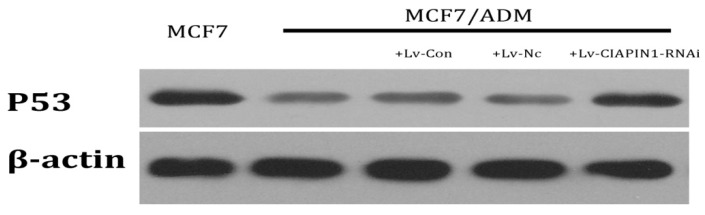
Expression of P53 Protein in Breast Cancer Cell Lines Before and After RNAi by Western blot.

We detected the influence of RNA interference targeting CIAPIN1 on P53 by Western blot. The results indicated that the protein expression of P53 in MCF7/ADM cell lines increased after RNAi which means that CIAPIN1 may participate in MDR of breast cancer by regulating P53 expression (Figure 10).

### 2.2. Discussion

As we have described, breast cancer is one malignant tumor which endangers the health of women. Many routine chemotherapies achieve poor therapeutic effects as well as bad prognosis, both of which cause huge clinical problems. It is believed that MDR is the key factor in the failure of breast cancer treatments. There are many complicated mechanisms for causing MDR [17,18,19,20,21]. Study on MDR reversal by drugs has been performed for over 20 years, however, many problems still need to be solved. Finding and studying genes related with MDR of breast cancer as well as their mechanisms for obtaining new targets which can improve therapeutic effects has become a current hot-spot for medical research. CIAPIN1 is a new apoptosis-regulating molecule independent of the bcl-2 family, and caspase family. It is produced by stimulation of many cell factors, which widely exist in all kinds of normal tissues of fetus and adult, especially in hematopoietic organs like the liver and spleen of fetus as well as in tissues with higher metabolism, where expression of CIAPIN1 is relatively high [22]. Although the physiological functions of CIAPIN1 have not been fully understood, however, current study has revealed that CIAPIN1 may have wide and important functions, especially its close correlations with malignant tumors. A primary study found that CIAPIN1 may protect mouse IL-3-dependent cell line Ba/F3 from etoposide and γ-rays [9]. In recent years, more studies also confirmed that CIAPIN1 participates in the MDR process of malignant tumor cells, but to date there are no studies on the effects of CIAPIN1 gene in MDR of breast cancer, therefore our study mainly focuses on this question and aims to understand effects of CIAPIN1 gene in MDR of breast cancer as well as its mechanisms looking forward to bringing new hopes for reversing MDR by gene therapy. 

First of all, we tested CIAPIN1 expression in breast cancer tissues and adjacent normal tissues from 41 patients by immunohistochemistry, and the results revealed a positive expression rate of CIAPIN1 (34.1%), which was significantly lower than that of normal group. This result is similar to results from studies on renal cell carcinoma [23] and lung cancer [24], indicating CIAPIN1 gene may be a tumor-suppressing gene and its down-regulating expression may be correlated with breast cancer. In our study the expression rate of CIAPIN1 had no correlations with age, tumor size, lymph node metastasis, TNM stage, pathological type, ER and PR expression, which is consistent with the results of the study of Hao on gastric cancer [12]. This indicates CIAPIN1 is not correlated with development and metastasis of breast cancer. Whether CIAPIN1 really participates in development and metastasis of breast cancer and its related mechanisms will be further studied. Owing to the fact P-gp plays a key role in MDR of breast cancer [25], we focused on studying the correlation between CIAPIN1 and P-gp, and the results revealed that P-gp expression in breast cancer was positively correlated with CIAPIN1 expression. Additionally we tested CIAPIN1 expression in many cell lines including MDA-MB-453, MDA-MB-231, MCF7, MCF7/ADM, ZR-75-30, SK-BR-3 by Western blot assays, the results of which indicated that the MCF7/ADM cell line with MDR characteristics had the highest CIAPIN1 expression compared to the parental MCF7 line. These studies primarily revealed that CIAPIN1 participates in MDR of breast cancer, and may be correlated with the MDR1 gene, which lays the foundations for our follow-up experiments.

RNA interference is post-transcriptional gene silencing mediated by double-stranded RNA with a specific sequence [26]. As a simple and effective technique taking the place of gene knockout, RNA interference is highly sequence-specific, highly effective, and fast-acting. Since 1998 when RNA interference was found, it had been widely used in studying gene functions and disease treatments [27]. Compared to artificial siRNA, shRNA with a stem-loop structure is more efficient and specific [28]. In our study we adopted lentiviral vector-mediated shRNA to inhibit expression of the CIAPIN1 gene. First of all, we used design software to analyze the CIAPIN1 full-length gene, and eventually three shRNA sequences were designed targeting its CDS region. Each sequence included two complementary single-stranded DNA templates, both of which were connected by 5'-CTTCCTGTCAGA-3', and followed by RNA PolyIII polymerase transcription termination sites (TTTTT) on the back, meanwhile BamHI and EcoR I restriction enzyme sites were added on both ends of the template strand separately. After this sequence was transferred into cells, RNA chain folded into shRNA with hairpin structure. These shRNA may be cut into siRNA by intracellular Dicer enzyme. Compared to chemical synthesis and *in vitro* enzymatic synthesis, expression vector may consistently produce siRNA by transcription within cells. Therefore the time of action of siRNA may be prolonged, which leads to silencing of the target gene. Additionally it is more reliable to select a stable cell line by lentiviral transfection. Owing to the fact lentivirus have almost no immunogenicity, therefore gene intervention by lentivirus should be one of the most effective methods [29]. Compared to injection of siRNA produced by chemical synthesis or plasmid DNA, it has more advantages, which are beneficial for further *in-vivo* study [30,31]. Our study adopted the third-generation lentiviral vector which had the least relativity with wild-type human HIV-1 virus, and this extremely increased the security of the vector. After we successfully transferred target gene into the purpose vector, lentivirus with no replicability was produced in 293TN cells to inhibit the CIAPIN1 gene. Results of Real-time PCR revealed that rate of CIAPIN1 gene silencing could reach up to 88%. Western blot results indicated that the level of protein expression also achieved corresponding results (over 83%). Additionally observation on different generations of cell cloning after infection by immunofluorescence found GFP expression did not change significantly with the increasing generation of cell clones. This demonstrated that expression of target gene in a stable cell line did not change with the increasing generation of cells. A series of tests revealed lentiviral transfection may effectively achieve RNA interference of the CIAPIN1 gene, and specifically inhibit CIAPIN1 gene expression in target cells. 

After successfully performing RNA interference on the CIAPIN1 gene, we studied the changes of cell resistance of an MCF7/ADM cell line towards chemotherapy by IC_50_ value changes. Doxorubicin, paclitaxel, gemcitabine were selected as drugs commonly used in treating breast cancer, all of which have MDR characteristics and MDR1 gene plays a vital role in their resistances, therefore they are often used in studies of MDR in breast cancer [32,33,34]. The results revealed that when CIAPIN1 expression was suppressed, the resistance of cell lines towards the three medicines were all significantly decreased, which further confirmed that CIAPIN1 gene played a vital role in MDR of breast cancer, and we could overcome resistance of breast cancer by down-regulating expression of the CIAPIN1 gene. Therefore which mechanisms CIAPIN1 gene may adopt to regulate MDR of breast cancer is also a highlight in our study. 

As mentioned above, the most important mechanism affecting MDR of breast cancer is drug resistance-associated proteins on the tumor cell membrane, especially P-gp encoded by MDR1. By the action of chemotherapy, expression of MDR1 on tumor cell membrane is up-regulated through many signal transduction pathways, and expression of P-gp which is encoded by MDR1 increases to protect tumor cells from apoptosis caused by chemotherapy [35]. Inhibiting MDR1 gene expression for the prevention of and to overcome MDR of breast cancer may improve response of chemotherapy, and prognosis of patients. This study adopted Real-Time PCR and Western blot tests to examine the expression of MDR1 and P-gp, the results of which revealed that when the CIAPIN1 gene in the MCF7/ADM cell line was down-regulated, expression of MDR1 mRNA and P-gp also decreased significantly. This indicated that CIAPIN1 gene may induce MDR of breast cancer by enhancing MDR1 gene. Additionally, P-gp may bind with rhodamine specifically [36], and can transfer rhodamine out of cells. This process is the same as P-gp transferring anti-tumor drugs. Rhodamine itself is fluorescent, which is easy to detect, therefore we often use Rhodamine efflux experiments to determine cells’ ability to pump drugs, and P-gp on cell membranes can be detected. Staining results revealed that the fluorescence intensity of MCF7/ADM decreased significantly compared to its parental MCF7, indicating the efflux function of resistant cells was relatively strong, whereas RNA interference may induce silencing of the CIAPIN1 gene, and staining fluorescence intensity increased significantly, which demonstrated that the efflux ability of cells had decreased. All results above further revealed that CIAPIN1 gene may affect MDR of breast cancer by regulating expression of MDR1. 

Abnormal proliferation is a basic characteristic of tumor cells, and the fundamental reason for uncontrolled proliferation lies in an imbalance of cell cycle regulation. Studies on the cell cycle of tumor cells have revealed that resistant cell lines have more S-phase cells, which promoted proliferation of tumor cells. Results from our study also found that MCF7/ADM cell line had more S-phase cells comparing to parental cells, but after RNA interference, G1-phase cells increased significantly with S-phase cells decreasing significantly (*p* < 0.001). This indicated that DNA synthesis was suppressed, and most cells were arrested in the G1 phase. Therefore we could confirm that CIAPIN1 may participate in MDR of breast cancer by regulating the cell cycle directly or indirectly. However there is a study finding that after CIAPIN1 gene expression was down-regulated by RNAi, S-phase cells increased significantly, which indicated that CIAPIN1 gene played the role of tumor suppressor gene and this was inconsistent with what we found [13]. This discrepancy can be explained by heterogeneity of tumor cells or other reasons that need further study. 

Apoptosis is regulated by apoptosis-related genes. Abnormal apoptosis not only plays a vital role in tumor development, but also participates in MDR of tumor cells and co-induces MDR through other pathways, which makes tumor cells resistant to apoptosis induced by chemotherapy [37,38,39]. Our study revealed that by the action of ADM, anti-apoptosis ability of MCF7/ADM cell line was pretty strong, however, after performing RNAi on CIAPIN1 gene, its anti-apoptosis ability was also identical with its parental cell line. The study found that Bcl-2 expression in some tumor cell lines was up-regulated with Bax and Fas expression down-regulating. However after CIAPIN1 gene was suppressed, Bcl-2 expression decreased with increasing Bax and Fas expression, therefore MDR of cells was arrested. Studies confirmed that apoptosis-related genes affected MDR of cells by regulating apoptosis. Whether CIAPIN1 affected MDR of cells through these genes or other apoptosis-related genes in breast cancer will be our next research focus. 

P53 plays a vital role during tumor development, and many studies have revealed that it participates in MDR. When P53 gene mutation, deletion occurs (Mutant p53), its regulation on apoptosis also becomes abnormal [40,41]. Apoptosis induced by chemotherapy is suppressed, which leads to significantly increasing resistance of tumor cells. Besides regulating apoptosis, P53 also participates in regulation of P-gp, and gene mutation may promote MDR of tumor cells through specific activation of MDR1/P-gp promoter. In our study we found that when CIAPIN1 gene expression was down-regulated, expression of wild-type P53 was up-regulated, which indicated that CIAPIN1 gene may down-regulate expression of wild-type P53. Therefore we assumed that CIAPIN1 gene may up-regulate MDR1 expression by regulating wild-type P53 expression, which leads to breast cancer cell resistance. Meanwhile we must emphasize that in our study we selected MCF7/ADM and its parental MCF7 cell line as targets, and whether this conclusion still holds for other breast cancer cell lines still needs further study [42]. 

## 3. Experimental

### 3.1. Tissue Samples, Cell Lines and Cell Culture

Forty-one breast cancer and adjacent benign tissue samples were obtained from patients who underwent surgical treatment at the Affiliated Tumor Hospital of Harbin Medical Hospital from August, 2009 to May, 2010. All samples were obtained from patients who gave informed consent to use excess pathological specimens for research purposes only. All patients had undergone no chemoradiotherapy or endocrine therapy preoperation. The multidrug resistant human breast carcinoma cell line MCF 7/ADM and along with their parental sensitive cell lines MCF7 were obtained from Shanghai Jiao Tong University and the CAS cell storeroom respectively. MCF7 and MCF7/ADM cells were cultured in DMEM (Invitrogen, Carlsbad, CA, USA) in a 5% CO_2_ incubator at 37 °C, supplemented with 10% fetal bovine serum (GIBCO, New York City, NY, USA) and antibiotics.

### 3.2. Design and Synthesis of Recombined Plasmid of siRNA Targeting CIAPIN1

The complementary DNA sequence of CIAPIN1 (NM_020313.2) was obtained from Genbank. The potential target sequences for RNA interference (RNAi) were scanned with the siRNA Target Finder and Design Tool available at the Ambion Web site (http://www.ambion.com/techlib/misc/siRNA_finder.html). The designed CIAPIN1 siRNA sequences are: 5'-GATCCGGAGCCAGTAGAGACAGCTCTTCCTGTCAGAAGCTGTCTCTACTGGCTCCTTTTTG-3'(CIAPIN1siRNA1), 5'-GATCCGTGGGTTCTTCTAGGCAGCCTTCCTGTCAGAGCTGCCTAGAAGAACCCACTTTTTG-3' (CIAPIN1siRNA2) and 5'-GATCCGTCAGCTTGTGGAAACTGCCTTCCTGTCAGAGCAGTTTCCACAAGCTGACTTTTTG-3'(CIAPIN1siRNA3). The forward and reverse sequences were synthesized, annealed and coloned into the BamHI/EcoR I of pSIH1-H1-copGFP vector (System Biosciences, San Francisco, CA, USA), yielding recombinant plasmids psiCIAPIN1-1, psiCIAPIN1-2 and psiCIAPIN1-3. The inserted sequences were confirmed by DNA sequencing. These recombinant plasmids and controls (empty plasmid and siRNA-Negative-plasmid) were transfected into 293 cell with Lipofectmaine^TM^ 2000 (Invitrogen) following the manufacturer’s instruction to determine the most efficient interfering sequence.

### 3.3. Recombinant Lentivirus Generation and Lentivirus Infection

Lentivirus Package plasmids mix (System Biosciences) and selected psiCIAPIN1-1 were cotransfected into 293TN cells to package and produce lentiviral vector and viral titer was determined according to the gradient dilution method. Lentivirus (1 × 10^4^ IFU/μL) packaging of green fluorescent protein (GFP) was infected into MCF7/ADM at various volume to determined the best MOI value at which concentrations no virus toxicity effect on cells was found. Then the experimental group (MCF7/ADM + CIAPIN1-siRNA1) and control group (MCF7/ADM + Lv-Negative) were added viruses at MOIs of 6 and incubated in normal condition. 96 h after infection we selected cells in two holes of which the GFP brightness is the strongest and proceeded intermediate clone culture in experimental group. 

### 3.4. Real Time-PCR Analysis

TRIZOL reagent (Invitrogen) was used to extract total RNA according to the protocol of the manufacturer. Reverse transcription was done by reverse transcriptase to synthesis cDNA. The polymerase chain reaction (PCR) primers used were as follows: As for CIAPIN1, 5'-CCTGTACGCCA ACACAGTGC-3' (forward), 5'-ATACTCCTGCTTGCTGATCC-3' (reverse) and for β-actin, 5'-CCTTCGGCCTGGTGGATGTCTTT-3' (forward), 5'-CTCCCGCTGCAGCTCTTTCACTTC-3' (reverse), while for MDR1, 5'-CCGTGGGGCAAGTCAGTTCA (forward), CCGGTCGGGTGGGAT AGTTG-3' (reverse) and for β-actin, 5'-CCTGTACGCCAACACAGTGC-3' (forward), 5'-ATACTCC TGCTTGCTGATCC-3' (reverse). PCR products were separated on a 1% agarose gel, visualized and photographed under ultra violet light. In relative quantitation measurement the date was analyzed by CT value compare to β-actin mRNA.

### 3.5. Western Blot Analysis

For protein analysis, the cell lysates were harvested and detected by western blots. The antibodies used were monoclonal antibodies against CIAPIN1 and P-gp (Abcam, Cambridge, UK), anti-P53 and anti-β-actin (Santa Cruz, CA, USA) antibodies, Anti-mouse HRP-linked Antibody (Santa Cruz) and Anti-rabbit HRP-linked Antibody (Cell Signaling Technology, Danvers, MA, USA). Densitometry analysis of protein levels was performed by using Scion-Image 4.0.2 software (Scion Corporation, Frederick, MD, USA).

### 3.6. Detect IC50 Values of Cells against Various Chemotherapeutics

Each group of cells (MCF7; MCF7/ADM; MCF7/ADM+CIAPIN1-RNAi) in logarithmic phase was seeded in culture plates of 96 wells at 37 °C, 5% CO_2_. Each well was added various chemotherapeutics (epirubicin, paclitaxel, gemcitabine) in different doses. OD values of the cells were tested by the method of MTT after 24 h incubation. Cell Growth inhibition ratio = (1 − average OD value of experimental group/average OD value of control group) × 100%. IC50 value was calculated. 

### 3.7. Cell Cycle Analysis

Standard fluorescence-activated cell sorter analysis was used to determine the distribution of cells in cell cycle. Briefly, cells in logarithmic phase were seeded in culture plates of 6 wells containing DMEM + 10% FBS at 37 °C, 5% CO_2_ with the final cell density of 1 × 10^5^ per well. Adherent cells then were collected by trypsinization and fixed with 70% ethanol overnight at 4 °C after 24 h culture. After washing with phosphate-buffered saline (PBS), the cells were added 100 μL PBS containing 100 μg/mL RNase A (Roche Diagnostics, Mannheim, Germany) and 50 μg/mL propidium iodide (Sigma, Santa Clara, CA, USA) and incubated for 30 min at room temperature. The samples were analyzed using a FACS flow cytometer (Becton Dickinson, San Jose, CA, USA). The cell cycle distribution was established by plotting the intensity of the propidium iodide signal, which reflects the cellular DNA content. Findings from at least 20,000 cells were collected and analyzed with the Cell Quest software (Becton Dickinson). 

### 3.8. Apoptosis Analysis

Apoptotic MCF7/ADM cells were quantified by annexin V-FITC staining, using a kit purchased from Becton Dickinson. In brief, cells in logarithmic phase were seeded in culture plates of 6 wells containing DMEM + 10% FBS for 24 h and DMEM without FBS for 8 h. Then cells were maintained in medium containing doxorubicin (5 μg/μL) at 37 °C, 5% CO_2_ for 24 h. Cells were washed twice with ice-cold PBS and then resuspended in 500 μL binding buffer at a concentration of 1 × 10^6^ cells/mL. 5 μL AnnexinV-FITC and 5 μL propidium iodide were added to these cells in order. The fluorescence was immediately determined by flow cytometry. Excitation wave length is 488 nm. Green fluorescence of Annexin V-FITC was detected by FITC channel l (FL1) while red fluorescence of PI (FL2) was detected by PI channel.

### 3.9. Rhodamine 123 Staining Test

We paved coverslipd treated with 37% sulphuric acid on the bottom of culture plates of 6 wells. Cell suspension (5 × 10^4^ cells/mL) was harvested after trypsinization and seeded in 6 wells and cultured for 24 h. Then we taken out coverslip and dropped Rh123 buffer (2 µM) on it. After 45 min incubation 4% paraform-aldehyde solution was then added. We observed the staining condition of cells using fluorescence inverted microscope. 

### 3.10. Statistical Analysis

All experiments were performed at least three times. Statistical analysis was performed using Student’s *t*-test by SPASS13.0 software and statistical significance was expressed as * *p* < 0.05.

## 4. Conclusions

MDR of breast cancer is an extremely complicated and consistently changing process, which is regulated by a series of genes and factors. In our study we confirmed that CIAPIN1 gene participates in this process, which was accomplished by regulation of MDR1 and P53 as well as influencing cell cycle and apoptosis. Meanwhile we also confirmed that performing RNAi on CIAPIN1 gene by a viral transfection approach may effectively reverse MDR of breast cancer, which provides a new way for improving therapeutic effects. This represents a promising gene therapy.

## Figures and Tables

**Table 1:** Relationship among the expression of P-gp, CIAPIN1 and P53 in breast cancer tissues.

P-gp	CIAPIN1			p53		
	+	−	P	+	−	P
+	11	10	<0.01	13	8	<0.025
−	3	17		4	16	

**Table 2:** IC50 values of MCF7/ADM cell line against various chemotheraputic drug before and after RNAi (μg/mL, $\bar{x}$ ± s).

Group	IC50 value		
	paclitaxel	epirubicin	gemcitabine
MCF7	0.808 ± 0.004	1.067 ± 0.016	5.859 ± 0.551
MCF7/ADM	7.121 ± 0.312	11.206 ± 1.789	49.724 ± 4.522
MCF7/ADM + CIAPIN1-RNAi	1.134 ± 0.057 *	4.514 ± 0.203 *	18.298 ± 1.273 *

* MCF7/ADM RNAi verse MCF7/ADM *p* ≤ 0.01.
